# miR-34a Regulates Mouse Neural Stem Cell Differentiation

**DOI:** 10.1371/journal.pone.0021396

**Published:** 2011-08-03

**Authors:** Márcia M. Aranha, Daniela M. Santos, Susana Solá, Clifford J. Steer, Cecília M. P. Rodrigues

**Affiliations:** 1 Research Institute for Medicines and Pharmaceutical Sciences, Faculty of Pharmacy, University of Lisbon, Lisbon, Portugal; 2 Department of Medicine, University of Minnesota Medical School, Minneapolis, Minnesota, United States of America; 3 Department of Genetics, Cell Biology, and Development, University of Minnesota Medical School, Minneapolis, Minnesota, United States of America; University of Nebraska Medical Center, United States of America

## Abstract

**Background:**

MicroRNAs (miRNAs or miRs) participate in the regulation of several biological processes, including cell differentiation. Recently, miR-34a has been implicated in the differentiation of monocyte-derived dendritic cells, human erythroleukemia cells, and mouse embryonic stem cells. In addition, members of the miR-34 family have been identified as direct p53 targets. However, the function of miR-34a in the control of the differentiation program of specific neural cell types remains largely unknown. Here, we investigated the role of miR-34a in regulating mouse neural stem (NS) cell differentiation.

**Methodology/Principal Findings:**

miR-34a overexpression increased postmitotic neurons and neurite elongation of mouse NS cells, whereas anti-miR-34a had the opposite effect. SIRT1 was identified as a target of miR-34a, which may mediate the effect of miR-34a on neurite elongation. In addition, acetylation of p53 (Lys 379) and p53-DNA binding activity were increased and cell death unchanged after miR-34a overexpression, thus reinforcing the role of p53 during neural differentiation. Interestingly, in conditions where SIRT1 was activated by pharmacologic treatment with resveratrol, miR-34a promoted astrocytic differentiation, through a SIRT1-independent mechanism.

**Conclusions:**

Our results provide new insight into the molecular mechanisms by which miR-34a modulates neural differentiation, suggesting that miR-34a is required for proper neuronal differentiation, in part, by targeting SIRT1 and modulating p53 activity.

## Introduction

MicroRNAs (miRNAs or miRs) are small, ∼21–23 nucleotide-long regulatory RNA molecules encoded in plant and animal genomes. miRNAs regulate the expression of target genes by binding to the 3′-untranslated regions of specific mRNAs and triggering mRNA destabilization or suppression of translation [1]. miRNAs appear to fine-tune gene expression by effecting more subtle and rapid changes than global transcriptional control mechanisms [2]. Each miRNA may regulate multiple genes; in mammals, miRNAs are predicted to control the activity of ∼50% of all protein-coding genes [3].

Functional studies indicate that miRNAs participate in the regulation of a number of cellular processes, including differentiation. The important regulatory role of miRNAs in development and differentiation clearly emerged from the study of embryonic stem cells null for the *Dicer* gene, which encodes an RNase III required for miRNA biogenesis. Ablation of *Dicer* affects embryonic stem cell division and proliferation [4], causing death in mice and complete loss of pluripotent stem cells [5]. In addition, *Dicer*-deficient embryonic stem cells also failed to differentiate. Therefore, miRNAs can influence stem cell fate at the levels of both proliferation and differentiation.

miR-34a is a member of the miR-34 family, which in mammals also includes miR-34b, and -34c [6]. miR-34a is encoded by its own transcript, whereas miR-34b and miR-34c share a common primary transcript. In mice, miR-34a is ubiquitously expressed with the highest expression in the brain [7], whereas miR-34b and c are mainly expressed in lung tissue [8]. Importantly, several reports showed that members of the miR-34 family are direct p53 targets, and their upregulation induces apoptosis and cell cycle arrest [8]–[13]. However, there is growing evidence to suggest a role for miR-34a during cell differentiation. miR-34a is involved in monocyte-derived dendritic cell differentiation by targeting *JAG1* [14]. In addition, miR-34a regulates phorbol ester-induced megakaryocytic differentiation of K562 cells by inhibiting cell proliferation and inducing cell cycle arrest, through direct regulation of its known targets cyclin-dependent kinase 4 and 6 (CDK4 and CDK6), MYB and mitogen-activated protein kinase kinase 1 (MEK1) [15]–[16]. Recently, miR-34a was shown to modulate mouse embryonic stem cell differentiation by regulating silent information regulator 1 (SIRT1) expression levels [17]. Nevertheless, the precise role of miR-34a during neuronal differentiation and the relevance of SIRT1 targeting have not been reported.

SIRT1 is a NAD-dependent deacetylase involved in cellular resistance to stress, metabolism, aging, and tumor suppression [18]–[21]. In mammals, SIRT1 function is mediated by its deacetylating activity not only on histones, but also on key transcription factors, such as p53 (*p53*), forkhead transcription factors (*FOXO*), p300 histone acetyltransferase, tumor suppressor protein p73 (*p73*), E2F transcription factor 1 (*E2F1*), DNA repair factor Ku antigen 70-kDa subunit (*Ku70*), nuclear factor κ-B (*NF-κB*), and androgen receptor (*AR*) (for review, see [22]). In addition, SIRT1 knockout mice demonstrated embryonic and postnatal developmental defects [23], suggesting that SIRT1 is important in embryonic development. Indeed, SIRT1 has been identified as a redox sensor in mouse embryonic fibroblasts and undifferentiated muscle cells, in which it controls proliferation, cell cycle arrest and differentiation [24]–[25]. SIRT1 was also reported to play a role in cellular differentiation of human keratinocytes [26], in mouse embryonic stem cell differentiation and hematopoiesis [27], and in differentiation of neural progenitors [28]–[29].

Here, we have identified the role of miR-34a in regulating mouse neural stem (NS) cell differentiation by mechanisms that involve SIRT1 downregulation and increased p53-DNA binding activity.

## Results

### miR-34a modulates the appearance of postmitotic neurons and neurite outgrowth

We have previously reported that induction of mouse NS cell differentiation results in a mixed cell population composed by both neuronal and glial cells, which in turn differentiate in a time-controlled fashion within 8 days [30]. In addition, we have shown that this differentiation process is associated with the modulation of pro-apoptotic miRNA expression, including miR-16, let-7a and miR-34a [31]. To determine the effect of miR-34a on neural differentiation, we explored the effect of miR-34a modulation on the percentage of neural progenitors (Nestin^+^), neuronal precursors (β-III Tubulin^+^), postmitotic neurons (NeuN^+^) and astrocytes (GFAP^+^). Pre- and anti-miR-34a, and respective controls, were transfected into differentiating mouse NS cells at 3 days.

**Figure 1:**
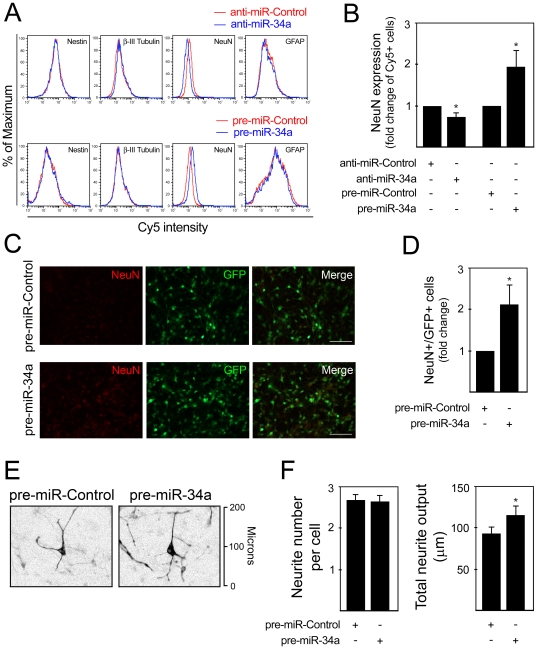
miR-34a modulates the proportion of NeuN-positive cells and neurite outgrowth. Mouse NS cells were transfected using 100 nM of either anti- or pre-miR-34a at 3 days, and collected after 24 and 72 h, respectively. Cells were subsequently labeled for Nestin, β-III Tubulin, NeuN and GFAP detection by flow cytometry. **A.** Representative histograms of Nestin, β-III Tubulin, NeuN and GFAP detection in anti-miR-control (red line) and anti-miR-34a-transfected cultures (blue line) (*top*), or in pre-miR-control (red line) and pre-miR-34a-transfected cultures (blue line) (*bottom*). In four independent experiments, similar alterations in the relative number of positive cells for each marker were observed. **B.** Quantification data of NeuN-positive cells assessed by flow cytometry after miR-34a modulation. **C.** Immunofluorescence detection of NeuN expression in 6 day cells transfected with pre-miR-Control and pre-miR-34a for 72 h. Scale bar, 50 µm. **D.** Quantification data of NeuN-positive cells assessed by immunocytochemistry after miR-34a modulation. **E.** Representative images of β-III Tubulin^+^ cells 48 h after pre-miR-transfection. Fluorescent images are shown in black and white and inverted for clarity. **F.** Neurite number and total neurite output are given to quantify the effect of miR-34a overexpression on cellular morphology. Neurites were manually traced using ImageJ v 1.43 and the NeuronJ plugin v 1.4.2. Results are shown as mean ± SEM. *p<0.05 from cells transfected with respective control.

Flow cytometry analysis revealed substantial alterations in NeuN expression, when cells were transfected with either anti- or pre-miR-34a (Fig. 1A). Notably, transfections with anti-miR-34a for 24 h reduced the proportion of NeuN^+^ cells by 26%, compared with cells transfected with an anti-miR negative control (*p*<0.05). Further, the percentage of NeuN^+^ cells significantly increased by 93% after pre-miR-34a transfection for 72 h (*p*<0.05) (Fig. 1B). The effect of miR-34a overexpression on the percentage of postmitotic neurons was also confirmed by immunocytochemistry, revealing increased number of NeuN^+^ cells after pre-miR-34a transfection (Fig. 1C and D). Modulation of miR-34a had no effect on the number of Nestin^+^ and β-III Tubulin^+^, while the effect on GFAP^+^ cells resulted in a slight tendency for a positive regulation. Importantly, cell counting and apoptosis assessment suggested that alterations in the number of NeuN^+^ cells were not related with altered total cell number or increased cell death (data not shown).

miR-34a overexpression also resulted in altered cellular morphology. To characterize this effect, neurite elongation and branching were assessed in β-III Tubulin^+^ cells 48 h after pre-miR-34a transfection (Fig. 1E). Although the number of neuritis was not changed, miR-34a overexpression led to a significant increase by ∼22% (*p*<0.05) in the length of neurites, suggesting that miR-34a modulates not only the appearance of postmitotic neurons but also plays a potential role in neurite elongation (Fig. 1F).

### SIRT1 expression is modulated by miR-34a during neural differentiation

**Figure 2:**
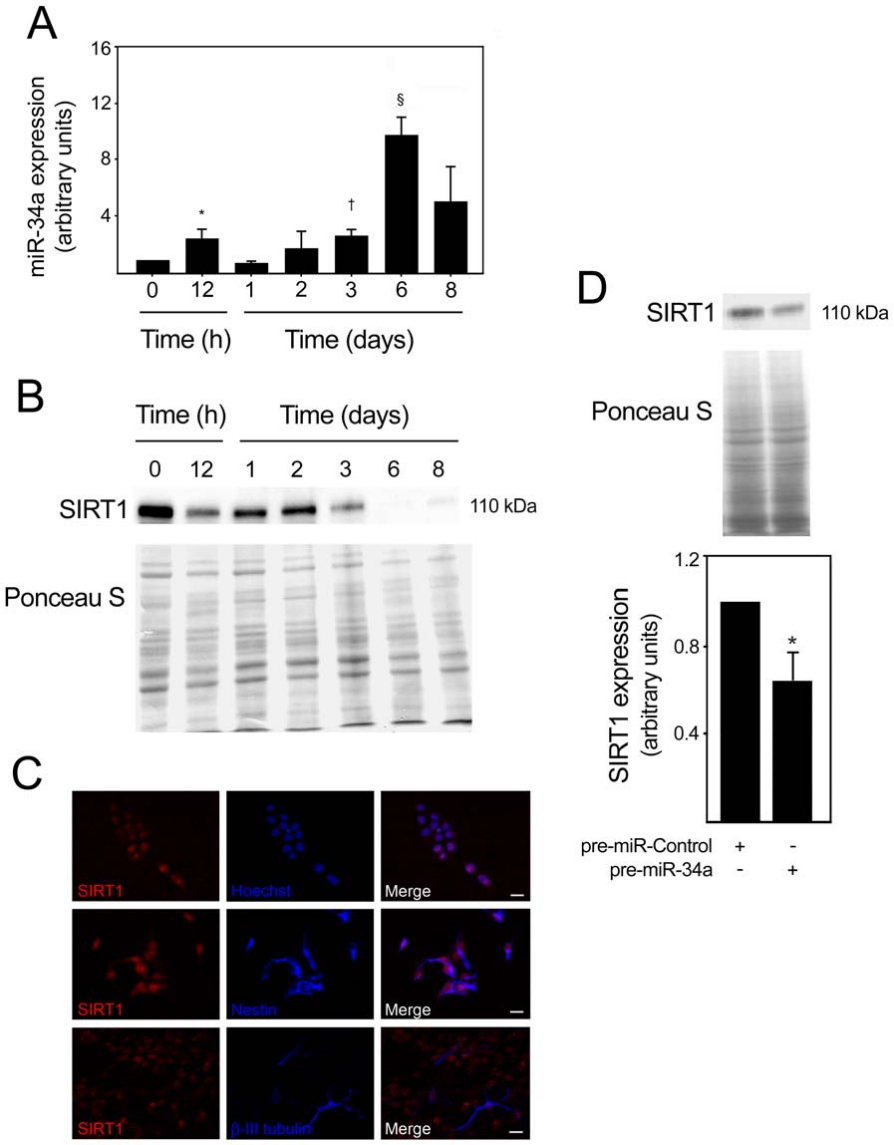
SIRT1 expression levels decrease after miR-34a overexpression. miR-34a expression was analyzed by quantitative Real Time-PCR using specific Taqman primers and GAPDH for normalization. Expression levels were calculated by the ΔΔCt method using undifferentiated cells as calibrator. SIRT1 expression in mouse NS cells was detected by immunoblotting at different times of differentiation or by immunocytochemistry at 2 days of differentiation. Cells transfected with either control or pre-miR-34a at 3 days of differentiation were also processed for SIRT1 detection by immunoblotting. **A.** Expression of miR-34a throughout the differentiation period. *p<0.05, ^†^ *p*<0.01 and ^§^ *p*<0.001 compared to day 0 (undifferentiated cells). Data represent mean ± SEM of four independent experiments. **B.** Representative immunoblot showing a marked decrease in SIRT1 expression levels from day 3 of differentiation. Ponceau S was used as loading control. **C.** Immunofluorescence detection of cells labeled with anti-SIRT1, anti-Nestin and anti-β-III tubulin antibodies to visualize nuclear SIRT1 expression in neural progenitors and neuronal precursors, respectively. Hoechst 33258 staining was used to visualize cell nuclei. Scale bar, 10 µm. **D.** Representative immunoblot (*top*) and corresponding densitometry analysis (*bottom*) showing decreased SIRT1 expression in pre-miR-34a transfected cells. Data represent mean ± SEM of three independent experiments. *p<0.05 from cells transfected with control.

Next, we investigated the mechanism by which miR-34a mediates neuronal differentiation. Several signaling pathways can be potentially affected by miR-34a, and implicated in the transition of dividing neuronal precursors to immature postmitotic neurons. SIRT1 has recently been suggested to be a target of miR-34a during embryonic stem cell differentiation [17]. In addition, SIRT1 expression was shown to be downregulated during differentiation of human embryonic stem cells [32]. When evaluating the expression of both miR-34a and SIRT1, we found that miR-34a expression was significantly upregulated from the third day on, increasing ∼11 fold (*p*<0.001) at day 6, when compared with undifferentiated cells (Fig. 2A). SIRT1 was shown to be highly expressed in undifferentiated cells and during the first two days of differentiation (Fig. 2B). Notably, SIRT1 expression markedly decreased from the third day of differentiation, which also corresponds with miR-34a upregulation. Immunofluorescence staining of SIRT1 at 2 days of differentiation revealed that SIRT1 staining was primarily nuclear and present in distinct cell populations, including Nestin^+^ and β-III Tubulin^+^ cells (Fig. 2C). Next, we evaluated whether overexpression of miR-34a resulted in decreased SIRT1 at the protein level. SIRT1 protein was significantly decreased after miR-34a overexpression (*p*<0.05) (Fig. 2D), suggesting that the effect of miR-34a on the number of postmitotic neurons and neurite elongation might be mediated by SIRT1.

### miR-34a may modulate neuronal outgrowth through a SIRT1-mediated mechanism

**Figure 3:**
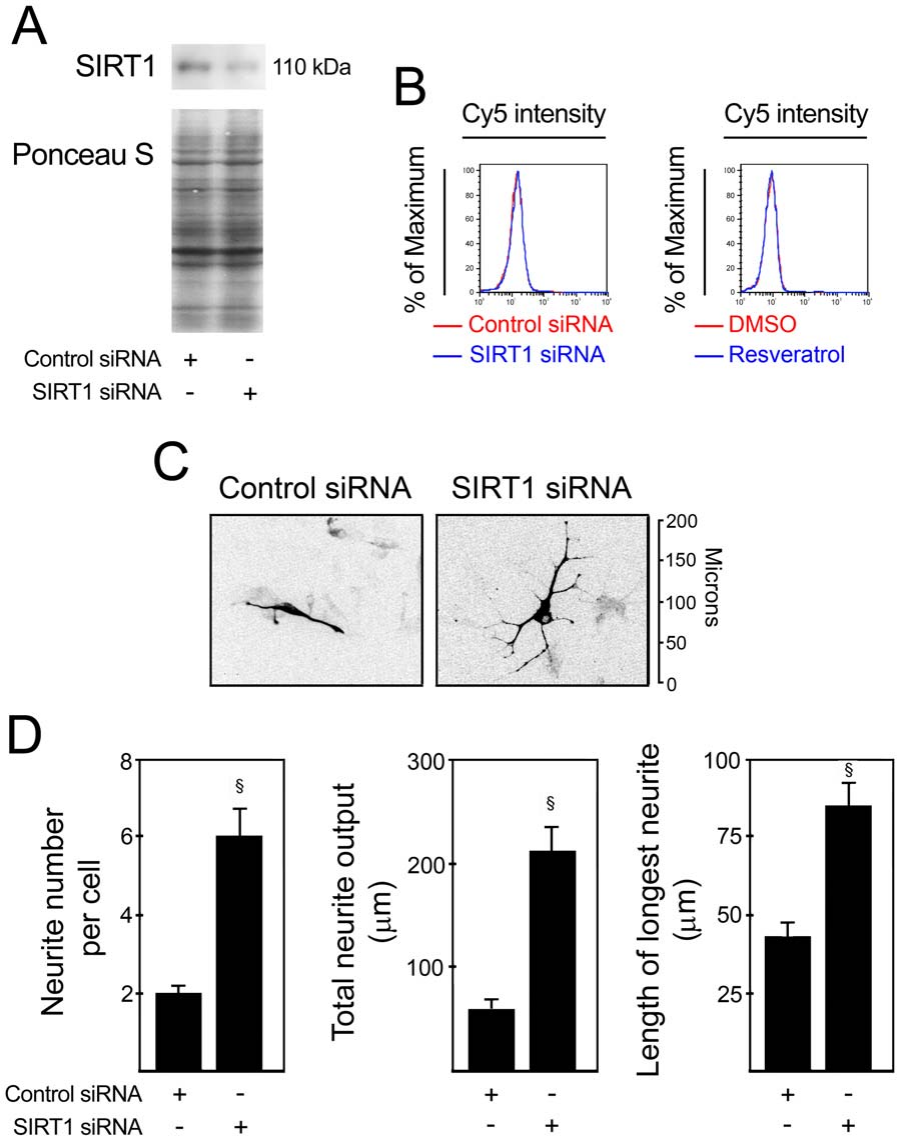
miR-34a may mediate neurite outgrowth by downregulating SIRT1 expression. SIRT1 expression was modulated in mouse NS cells at 12 h after induction of differentiation. Inhibition of SIRT1 was achieved by transfecting cells with either scrambled control or 100 nM of SIRT1 siRNA, while activation of SIRT1 was performed by treating cells with either DMSO (control) or 5 µM of resveratrol. Cells were collected at 3 days, fixed and processed for NeuN labeling and detection by flow cytometry or for immunocytochemistry of β-III Tubulin and subsequent characterization of neurite elongation and branching. The blue line corresponds to SIRT1 modulation, while the red line corresponds to the respective control. **A.** Representative immunoblot showing decreased SIRT1 expression after transfection with SIRT1 siRNA. Ponceau S was used as loading control. **B.** SIRT1 modulation had no effect on the percentage of NeuN^+^ cells. **C.** Representative fluorescence microscopy image of β-III Tubulin immunocytochemistry after SIRT1 downregulation. Fluorescent images are shown in black and white and inverted for clarity. **D.** Neurite number, total neurite output and the length of longest neurite were determined to quantify the effect of SIRT1 downregulation on cellular morphology. Neurites were manually traced using ImageJ v 1.43 and the NeuronJ plugin v 1.4.2. Results are shown as mean ± SEM. ^§^ *p*<0.0001 from cells transfected with respective control.

To confirm that SIRT1 participates in miR-34a-mediated neural differentiation, we initially determined whether SIRT1 modulation affects the percentage of postmitotic neurons. To address this question, cells were either incubated with resveratrol, a pharmacologic SIRT1 activator, or transfected with SIRT1 siRNA, at 12 h after incubation in differentiation medium. Changes in percentage of NeuN^+^ cells were evaluated by flow cytometry at 3 days of differentiation. The efficiency of SIRT1 silencing was confirmed by immunoblotting at 24 h after transfection (Fig. 3A). Our results showed that both SIRT1 silencing and SIRT1 activation had no effect on the percentage of NeuN^+^ cells (Fig. 3B). Similar results were obtained when evaluating the percentage of MAP2^+^ cells by immunocytochemistry after SIRT1 silencing (data not shown). These results suggested that the effect of miR-34a on the percentage of postmitotic neurons was not mediated by SIRT1.

Next, we investigated whether SIRT1 could mediate miR-34a effect on neurite outgrowth. For that, we analyzed neurite elongation and branching after downregulation of SIRT1 (Fig. 3C). Our results showed that β-III Tubulin^+^ cells exhibited a significant increase in neurite number after SIRT1 knockdown by ∼3-fold (*p*<0.0001). Similarly to miR-34a overexpression, transfection with SIRT1 siRNA also resulted in significantly increased total neurite output by ∼3.5-fold (*p*<0.0001) (Fig. 3D). These results support the observation that miR-34a may modulate neurite outgrowth by targeting SIRT1.

### miR-34a upregulation increases acetylation of p53 and p53-DNA binding activity

**Figure 4:**
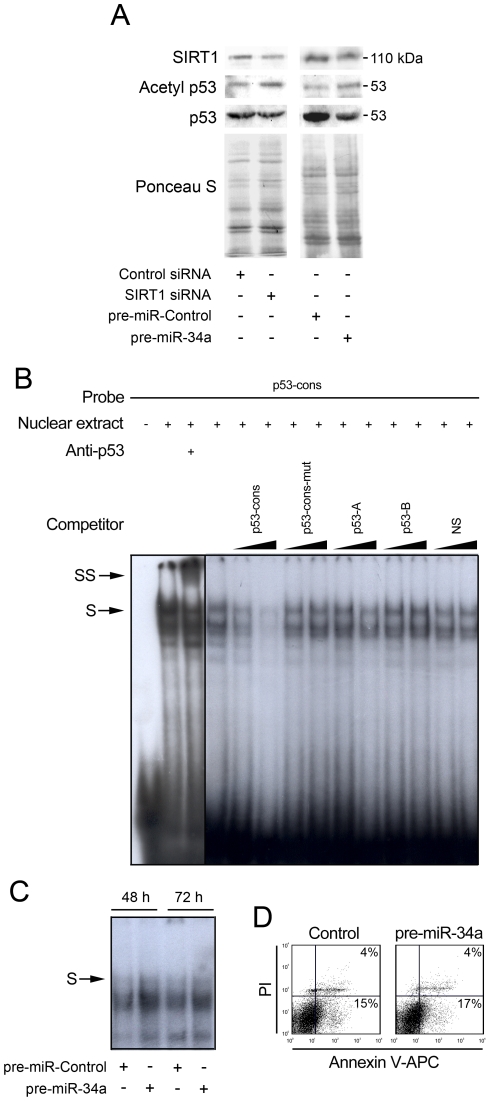
miR-34a overexpression increases acetylation of p53 and p53-DNA binding activity. Mouse NS cells at 3 days of differentiation were transfected with either SIRT1 siRNA or scrambled control for 48 h or with pre-miR-control or pre-miR-34a for 72 h. Cells were then collected for total and nuclear protein extraction or stained with Annexin-V-APC/PI to evaluate cell death. p53-overexpressing cells were used for supershift and competition experiments. Radio-labeled double-stranded oligonucleotide corresponding to the p53 *consensus* (p53 -cons) was used as a probe. **A.** Representative immunoblot showing increased p53 acetylation at lysine 379 after SIRT1 silencing or overexpression of miR-34a. Ponceau S was used as loading control. **B.** Representative EMSA showing the specificity of the complex formed with p53-cons probe. Supershift experiments were performed using an anti-p53 antibody (DO-1, Santa Cruz Biotechnology). Competition experiments were performed by adding 10- or 100-fold excess of unlabeled double-stranded oligonucleotides bearing a mutation in the consensus site (p53-cons-mut), or containing either two or one quarter-sites known to be consensus sites for p53 (p53-A and p53-B, respectively) or a non-specific sequence (NS). **C.** EMSA showing increased p53-DNA binding activity in pre-miR-34a transfected cells for 48 and 72 h. **D.** Representative Annexin V-APC/PI staining showing absence of cell death after miR-34a modulation.

SIRT1 has been shown to interact with and deacetylate the p53 protein, leading to a suppression in its activity [33]. The induction of miR-34a during mouse NS cell differentiation and the concomitant decrease of SIRT1 suggest that p53 may be involved in this regulation. Thus, we examined the effect of miR-34a on acetylation of p53. Initially, we confirmed that SIRT1 regulates acetylation of p53 by transfecting mouse NS cells with siRNA to knockdown SIRT1 and then measuring acetylated p53. Our results confirmed that knockdown of SIRT1 increased p53 acetylation at Lys 379 (Fig. 4A). Importantly, a similar increase in acetylated p53 was observed after miR-34a overexpression, suggesting that miR-34a may increase p53 acetylation and subsequent p53 transcriptional activity due to decreased SIRT1 expression.

To further explore this hypothesis, we upregulated miR-34a expression levels and evaluated p53-DNA binding activity by EMSA. To verify the specificity of the complex(es) formed when a p53 *consensus* oligonucleotide (p53-cons) was used as a probe, we performed a supershift assay using an anti-p53 antibody, as well as competition assays using oligonucleotides with different affinities for p53 binding, in mouse NS cells overexpressing p53. As shown in Fig. 4B, nuclear extracts from mouse NS cells overexpressing p53 exhibited very strong DNA-binding activity. In the presence of the p53 antibody, there was a shift of the specific complex, resulting in slower-migrating bands (Fig. 4B, arrowheads). Competition assays using 10 and 100-fold molar excess of each competitor resulted in reduced efficiency of DNA binding, according to their affinity for p53. In fact, competitor p53-A contains two quarter-sites known to be consensus sites for p53 [34], while p53-B contains only one (see Table S1). Therefore, the efficiency of competition was proportional to the number of repeats. As expected, competition with unlabeled p53-cons resulted in a significant decrease in the efficiency of DNA binding, and this effect was completely abolished when using p53-cons bearing a mutation in the consensus site. Indeed, the formation of p53/p53-cons complex was not competed by an unrelated DNA sequence. Importantly, the EMSA results show that total nuclear proteins capable of binding to the p53-cons probe markedly increase under miR-34a overexpression (Fig. 4C). These results suggested that miR-34a overexpression increased p53-DNA binding activity. Notably, this was not associated with increased cell death, as evaluated by PI/Annexin staining (Fig. 4D).

### Astrogliogenesis can be modulated by miR-34a through a SIRT1-independent mechanism

**Figure 5:**
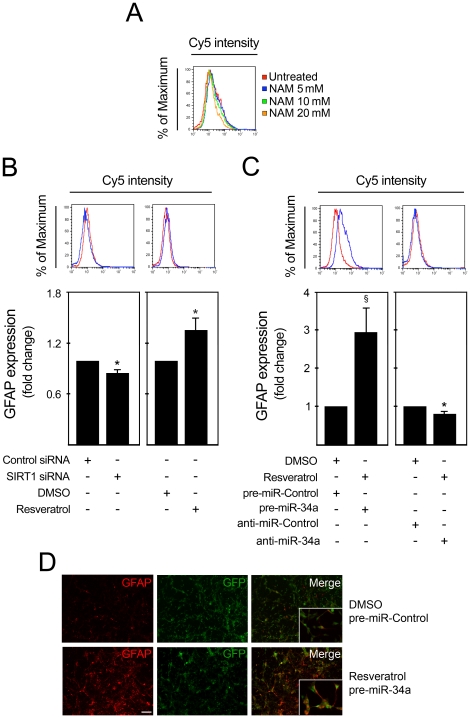
miR-34a promotes astrocytic differentiation under resveratrol treatment. SIRT1 expression was modulated in mouse NS cells after 12 h of induction of differentiation. Inhibition of SIRT1 was achieved by incubation with nicotinamide or by transfecting cells with either scrambled control or 100 nM of SIRT1 siRNA. SIRT1 was activated by treating cells with either DMSO (control) or 5 µM of resveratrol. Cells were collected at 3 days, fixed and processed for GFAP labeling and detection by flow cytometry and immunofluorescence. The blue line corresponds to SIRT1 modulation, while the red line represents the respective control. **A.** Incubation with nicotinamide, decreased the percentage of GFAP^+^ cells in a dose-dependent manner. **B.** Decreased percentage of GFAP^+^ cells after silencing of SIRT1 (*left*), and increased percentage of GFAP^+^ after SIRT1 activation (*right*). **C.** Under resveratrol treatment, overexpression of miR-34a resulted in increased proportion of GFAP^+^ cells (*right*), while miR-34a downregulation had the opposite effect (*left*). **D.** Immunofluorescence showing increased number of GFAP^+^ cells under resveratrol treatment and miR-34a overexpression (*bottom*) when compared with controls (*top*). Scale bar, 80 µm. Images taken with a magnification of 400× are shown in detail. *p<0.05 and ^§^ *p*<0.001 from cells transfected with respective control. NAM, nicotinamide.

Although transient transfection of miR-34a into mouse NS cells had a minor effect on astroglial subpopulation, we investigated whether SIRT1 modulation could affect astroglial differentiation. For this purpose, we modulated SIRT1 expression in cells at 12 h of differentiation by incubation with either SIRT1 inhibitor, nicotinamide; SIRT1 activator, resveratrol, or transfection with SIRT1 siRNA. Changes in the percentage of GFAP^+^ cells were monitored 36 h after modulation by flow cytometry. Incubation with the SIRT1 inhibitor, nicotinamide, led to a concentration-dependent reduction in the percent GFAP^+^ cells (Fig. 5A). In addition, SIRT1 silencing consistently decreased the number of GFAP^+^ cells by ∼15% (*p*<0.05), while activation of SIRT1 increased GFAP^+^ cells by ∼36% (*p*<0.05) (Fig. 5B), indicating that SIRT1 expression positively modulates astrocytic differentiation. Interestingly, under resveratrol treatment, overexpression of miR-34a resulted in a significant increase in GFAP^+^ cells by ∼3 fold (*p*<0.001) (Fig. 5C), while transfection with anti-miR-34a under the same conditions resulted in a decrease in GFAP^+^ cells by ∼17% (*p*<0.05). Immunofluorescence analysis of GFAP after pre-miR-34a transfection corroborated this observation (Fig. 5D). Therefore, resveratrol treatment further enhanced upregulation of astrocytic differentiation after miR-34a modulation. As miR-34a negatively regulates SIRT1 expression, we would have expected to have an opposite effect of miR-34a on GFAP^+^ cells. Thus, our results indicate that SIRT1 positively regulates the astrocytic subpopulation. Moreover, under certain conditions, miR-34a may upregulate astrocytic differentiation through a SIRT1-independent mechanism.

## Discussion

miR-34a is a well-known miRNA involved in various cellular functions that acts as a tumor suppressor [9], [11]. In this study, we demonstrated a novel function for miR-34a during neuronal differentiation, implicating its expression in the appearance of postmitotic neurons and on the regulation of neurite elongation via SIRT1 downregulation. In addition, its function was also associated with increased levels of acetylated p53 and p53-DNA binding activity, in common with neural differentiation.

We previously determined that miR-34a expression was concomitant with the appearance of postmitotic neurons and astrocytes during mouse NS cell differentiation [31]. Here we show that miR-34a promoted the appearance of postmitotic neurons. This is in agreement with previous observations that miR-34a can suppress cell-cycle genes and induce a neural phenotype [35]. miR-34a may have a wide range of molecular targets during cell differentiation. Our results showed that the specific effect of miR-34a on the appearance of postmitotic neurons was not mediated by SIRT1. Rather, miR-34a-targets, such as CDK4 and CDK6, as well as MEK1, which regulate cell cycle progression and inhibit cell proliferation, may constitute potential mediators of miR-34a effect on postmitotic neurons. In addition, miR-34a can also regulate Notch-1 and Notch-2 protein expression [36], which could contribute to the observed final outcome.

Here we report an additional effect of miR-34a on neurite elongation. Specifically, overexpression of miR-34a resulted in increased total neurite output. The fact that a similar phenotype was observed after SIRT1 knockdown suggests that this effect might be mediated by SIRT1. However, a recent publication reported that miR-34a expression was not involved in SIRT1 downregulation during human embryonic stem cell differentiation [32]. In fact, the authors observed no increase in miR-34a expression after induction of differentiation into embryonic bodies. Differences associated with specific properties of each biological model may explain this discrepancy; in addition to different mechanisms that regulate SIRT1 expression levels. Moreover, the effect of siRNA-mediated silencing of SIRT1 on neurite number/output was more pronounced than the effect of miR-34a overexpression. A possible explanation may rely on the fact that miRNAs usually function as a fine-tuning mechanism, and not as an on/off switch. In this scenario, siRNA transfection would result in increased SIRT1 downregulation when compared with miR-34a overexpression.

SIRT1 expression was shown to decrease during mouse NS cell differentiation. A similar pattern of expression was already reported for the differentiation of human embryonic stem cells [32], suggesting that decreased SIRT1 expression is a prerequisite for successful differentiation. In accordance, SIRT1 downregulation was shown to be necessary for reactivation of key developmental genes, such as the neuroretinal morphogenesis effector delta-like protein 4 (DLL4), the T-box transcription factor (TBX3), and paired box gene 6 (PAX6) [32].

Our observation that SIRT1 is expressed in β-III Tubulin^+^ cells at two days of differentiation suggest that SIRT1 is may be necessary in the initial stages of the differentiation process. In agreement, SIRT1 has been shown to positively regulate neuronal differentiation by inhibiting the transactivation of Hes1, a transcription factor that negatively controls cell differentiation [28]. In addition, loss of SIRT1 in embryonic stem cells was shown to delay the switch-off of *Oct4* and *Nanog* expression, thereby reducing the capacity to exit the pluripotent stem cell program [27]. This suggests that a tight regulation of SIRT1 expression might exist and that timing and levels of expression may determine commitment toward a certain neural phenotype.

miR-34a appears to be a key player parting the p53 regulatory network [37]. miR-34a transcription is directly activated by p53, and in turn miR-34a regulates the expression of some p53 target genes [9], [11], [12]. Nevertheless, transfection with p53 siRNA did not affect miR-34a expression, suggesting that induction of miR-34a during mouse NS cell differentiation is p53 independent (data not shown). These results are in accordance with previous data showing a p53-independent role for miR-34a during megakaryocytic differentiation of K562 cells [16]. Instead, p53 seems to act downstream of miR-34a in this cellular context. Our results suggested that miR-34a indirectly regulates p53, possibly through a SIRT1-dependent mechanism. Numerous studies indicated that SIRT1 plays a crucial role in p53-mediating responses by deacetylating human p53 at lysine 382 (K379 in mouse p53) in the C-terminal domain, which attenuates its transcriptional activity [38], [39]. Based on this, downregulation of SIRT1 mediated by miR-34a would result in increased levels of acetylated p53. The acetylated form has been reported to have increased transcriptional activity, and promote coactivator recruitment and site-specific DNA binding [40].

In fact, p53 was previously shown to be necessary for neurite outgrowth and axonal regeneration, which required specific acetylation of p53 [41]. Further, SIRT1-mediated regulation of p53 might also contribute to the induction of differentiation, since SIRT1 was shown to inhibit p53-mediated suppression of *Nanog* expression, which is required to maintain cells in an undifferentiated status [42]. Several reports support a role for p53 in neural differentiation [43]–[45], although the precise mechanism(s) remain unclear. It may involve the ability of p53 to promote cell cycle arrest by inducing *p21* expression [46]. Alternatively, p53 could directly regulate the transcription of neuronal genes. By genome-wide chromatin immunoprecipitation (ChIP), new putative p53 target genes have been identified during NGF-mediated PC12 neuronal differentiation. These include *wnt7b*, which is involved in dendritic development, and the *tfcp214*/grhl3 grainyhead homolog, implicated in ectodermal development [47]. Further, p53 regulates the expression of both actin-binding protein Coronin 1b and the GTPase Rab13, which are required for physiological neurite outgrowth in PC12 cells and dorsal root ganglion neurons [41]. Nevertheless, while the present study provides a foundation for the role of miR-34a in neural stem cell differentiation, the role of p53 in this pathway remains unclear.

Our results also demonstrate that SIRT1 expression positively regulates astrogliogenesis. In fact, it has been previously shown that increased SIRT1 activity causes differentiation of neural progenitor cells into astrocytes at the expense of neurons [29]. SIRT1 represses the pro-neuronal basic helix-loop-helix (bHLH) transcription factor Mash1 and influences cell-fate decision. This indicates that SIRT1 might play a distinct role on differentiation under specific conditions, according to substrate and co-factor availability, as well as redox state of the cell.

We found that transient transfection of miR-34a into mouse NS cells had almost no effect on the astrocytic subpopulation. This is in accordance with previous studies showing that miR-34a overexpression did not affect human astrocytes [36]. Curiously, miR-34a was shown to positively regulate the proportion of GFAP^+^ cells when cells were pre-treated with resveratrol. Given that miR-34a negatively regulates SIRT1 expression and SIRT1 induces the astroglial lineage, we would have expected an opposite effect of miR-34a in GFAP^+^ cells. This suggests that under certain conditions, miR-34a may have the capacity to influence commitment toward astrogliogenesis by a SIRT1-independent mechanism. This data might be particularly important in the specific case of gliomas, since miR-34a has been extensively studied as a therapeutic target for tumor suppression.

It is thought that miRNAs may act as a buffering system, modulating molecules whenever cell balance is lost. Recent findings indicate that simple inactivation of miRNAs is not sufficient to collapse the signaling networks in which miRNAs are involved, and suggest that the use of sensitized backgrounds provides an efficient approach to study miRNA function analysis [48]. Therefore, it is possible that the particular environment generated by resveratrol treatment would render the cells sensitive to the modulation of miR-34a, revealing an additional miR-34a function, i.e. the capacity to regulate the glial lineage. Resveratrol is a naturally occurring polyphenol with antioxidant, anti-inflammatory, anti-aging, cardioprotective, and neuroprotective activities [49]. Multiple biochemical and molecular actions seem to contribute to its effects including inhibition of cyclooxygenase (COX) [50], cytochromes P450, cell invasion, transformation, and angiogenesis [51].

Resveratrol has been shown to up-regulate antioxidant enzymes such as glutathione peroxidase, catalase, and quinone reductase. It inhibits lipid peroxidation, ornithine decarboxylase (ODC), protein kinases, and cellular, proliferation [52] and was shown to inhibit inflammatory processes, including NF-κB activation and inflammatory gene expression [53], [54]. Resveratrol effectively induces apoptosis modulated through multiple pathways including up-regulation of p53, activation of caspases, modulation of Bcl-2 family proteins, inhibition of D-type cyclins, and interference with NF-κB and AP-1 mediated cascades [55]. Therefore, multiple targets could contribute to its effects in astrogliogenesis induced by miR-34a. Additional studies, possibly using genetic backgrounds with reduced activity of regulatory pathways modulated by resveratrol would be necessary to confirm the capacity and the mechanism of miR-34a to regulate astrogliogenesis.

In conclusion, our results support a role for miR-34a in the regulation of neural differentiation. In addition, miR-34a-mediated silencing of SIRT1 may be necessary for correct establishment of specific differentiation programs during neural stem cell differentiation.

## Methods

### Cell Culture

Mouse NS cells containing a constitutively expressed marker for green fluorescent protein (GFP) were used to investigate the process of neural differentiation. Primary cells were obtained from central nervous system tissue of embryonic mice, and cultured primarily as previously described [56]–[58]. Mouse NS cells were maintained as neurospheres in undifferentiation medium, serum-free, 1∶1 mix of DMEM/F12 (Invitrogen Corp., Grand Island, NY) with 1× N-2 supplement (Invitrogen Corp.), 20 ng/ml epidermal growth factor (EGF) (R & D Systems Inc., Minneapolis, MN), 20 ng/ml basic fibroblast growth factor (bFGF) (PeproTech EC, London, UK), and 1% penicillin-streptomycin (Invitrogen Corp.), at 37°C in humidified atmosphere of 5% CO_2_. Subculture occurred at day 7 with mechanical dissociation of neurospheres. The differentiation of mouse NS cells *in vitro* was induced by culturing dissociated cells in differentiation medium containing DMEM/F12 with 1× N-2 supplement, 100 ng/ml bFGF, 10% fetal bovine serum (FBS) (Invitrogen Corp.), 500 nM all-*trans* retinoic acid (Sigma Chemical Co., St. Louis, MO), 50 µM taurine (Sigma Chemical Co.), 10 ng/ml transforming growth factor-β2 (TGF-β2) (R & D Systems Inc.) and 1% penicillin-streptomycin in tissue culture plates pre-coated with poly-D-lysine (Sigma Chemical Co.). The culture medium was changed every 3 days. Differentiated cells at 5×10^5^ cells/ml were processed for immunobloting assays. Transfections and cell treatments were carried out at 2.5×10^5^ cells/ml cellular density.

### Small interfering RNA transfections, overexpression/antisense experiments and cell treatments

miR-34a expression was modulated using 100 nM of pre/anti-miR negative control and pre/anti-miR-34a (Applied Biosystems, Foster City, CA). The sequences of small interfering RNA (siRNA) duplex for SIRT1 were sense 5′- GUGAGAAAAUGCUGGCCUA-3′ and antisense 5′- UAGGCCAGCAUUUUCUCAC-3′. Cells were transfected using 100 nM of SIRT1 siRNA or treated with 5, 10 and 20 mM of nicotinamide or 5 µM of resveratrol or 0.1% DMSO (control) (Sigma Chemical, Co.). Mouse NS cells were transfected using Lipofectamine 2000 (Invitrogen Corp) following manufacturer's instructions. Efficiencies of miR-34a modulation and SIRT1 silencing were assessed by quantitative real-time RT-PCR and immunoblotting, respectively.

### Evaluation of miR-34a expression levels by quantitative Real Time-PCR

Real-time PCR was performed in an Applied Biosystems 7300 Sequence Detection System (Applied Biosystems, Foster City, CA). Ten ng of total RNA isolated by TRIzol reagent (Invitrogen Corp.) were reverse transcribed using a TaqMan® MicroRNA Reverse Transcription (RT) kit from Applied Biosystems. Each RT reaction contained 1× stem-loop RT specific primer, 1× reaction buffer, 0.25 mM each of dNTPs, 3.33 U/µl Multiscribe RT enzyme and 0.25 U/µl RNase inhibitor. The 15-µl reactions were incubated for 30 min at 16°C, 30 min at 42°C, and 5 min at 85°C and then held at 4°C. The PCR reaction was performed using a standard TaqMan® PCR kit protocol (Applied Biosystems). Briefly, following the RT step, 1.33 µl of the RT reaction were combined with 1 µl of a TaqMAn MicroRNA Assay (20×; forward primer, reverse primer and probe) and 17.67 µl of TaqMan® Universal PCR Master Mix, No AmpErase® UNG in a 20 µl final volume. The reactions were incubated at 95°C for 10 min, followed by 40 cycles of 95°C for 15 s and 60°C for 1 min; and were run in triplicate. miRNA expression levels relative to GAPDH were calculated on the basis of ΔΔ*C*t method. The n-fold change in miRNAs expression was determined according to the method of 2^−ΔΔCT^.

### Flow cytometry analysis of Nestin, β-III Tubulin, NeuN and GFAP

Cells were trypsinized (0.025% trypsin/EDTA; Invitrogen Corp.) and harvested in Ca^2+^-free and Mg^2+^-free PBS and 2% FBS. After washing, 0.5–1×10^6^ cells were fixed with paraformaldehyde (4% w/v) in PBS for 20 min on wet ice, and blocked for 20 min in PBS, containing 0.25% saponin (Fluka, Biochemika, Switzerland) and 5% normal donkey serum (Jackson ImmunoResearch Laboratories, Inc., West Grove, PA). Subsequently, cells were incubated with antibodies reactive to Nestin (MAB 353), neuronal nuclei (NeuN) (MAB377), glial fibrillary acidic protein (GFAP) (MAB360; Chemicon International, Temecula, CA ) and β-III Tubulin (Tuj1) at a dilution of 1∶100, 1∶20, 1∶2000 and 1∶500, respectively, in PBS containing 0.1% saponin and 5% normal donkey serum, for 30 min. Cells were then incubated with anti-mouse antibody conjugated to Cy5 (Jackson ImmunoResearch Laboratories, Inc.), for 30 min. Cells were analyzed using a FACSCalibur (Becton Dickinson, Mountain View, CA).

### Immunoblotting

Steady-state levels of SIRT1, p53 and acetylated p53 at lysine 379 were determined by immunoblotting. Total protein extracts of mouse NS cells were prepared in lysis buffer, following standard protocols. Protein content was measured by the Bio-Rad protein assay kit according to the manufacture's specification, using bovine serum albumin as standard. 50–80 µg of total protein extracts were separated on 12% sodium dodecyl sulphate-polyacrylamide electrophoresis gel and then subjected to immunoblots using primary mouse monoclonal antibody reactive to p53 (DO-1, SantaCruz Biotechnology) or rabbit polyclonal antibody reactive to SIRT1 (07-131, Chemicon International) and to acetyl-53 (Lys379) (2570, Cell Signaling). Blots were subsequently incubated with secondary antibodies conjugated with horseradish peroxidase (Bio-Rad Laboratories, Hercules, CA, USA). Finally, membranes were processed for protein detection using Immobilon (Millipore Corporation, Billerica, MA) or SuperSignal reagent (Pierce, Rockford, IL). Ponceau S staining was used to assess equal gel loading [59].

### Immunocytochemistry

Mouse NS cells were fixed with paraformaldehyde (4% w/v) in phosphate-buffered saline (PBS) and blocked for 1 hour at room temperature in PBS containing 0.1% Triton-X-100, 1% FBS, and 10% normal donkey serum (Jackson ImmunoResearch Laboratories, Inc., West Grove, PA). Subsequently, cells were incubated with either anti-SIRT1 (rabbit, polyclonal), anti-Nestin, anti-β-III Tubulin, anti-NeuN or anti-GFAP (mouse, monoclonal) antibodies (Chemicon International) at a dilution of 1∶200, 1∶100, 1∶500, 1∶20 and 1∶1000, respectively, in blocking solution, overnight at 4°C. Cells were then incubated with either anti-mouse or anti-rabbit IgG conjugated to AMCA (Jackson ImmunoResearch Laboratories, Inc.) and Alexa 594 (Molecular Probes-Invitrogen), respectively, for 2 h at room temperature. For NeuN and GFAP detection, an Alexa 568-conjugated anti-mouse antibody (Molecular Probes-Invitrogen) was used. In selected experiments, mouse NS cell nuclei were subsequently stained with Hoechst 33258 (Sigma Chemical Co.) at 50 µg/ml in PBS, for 5 min at room temperature. Samples were mounted using Fluoromount-G™ (Beckman Coulter, Inc.). Fluorescence microscopy assessments were performed with a Zeizz AX10 microscope (Carl Zeiss, Jena, Germany) equipped with a Leica DFC490 camera (Leica Weitzlar, Germany).

### Analysis of neurite outgrowth

Neurite number, total neurite output and the length of longest neurite were determined to evaluate the effect of SIRT1 silencing and miR-34a overexpression on neurite outgowth. For this purpose, cells were fixed at 3 and 6 days, for SIRT1 and miR-34a modulation, respectively, and stained with the marker β-III Tubulin to identify neuronal precursor cells. Neurites of labeled cells were manually traced using ImageJ v1.43 and the NeuronJ plugin v 1.4.2 [60]. For each measurement, data represent the average of ∼50 different neurons in each experiment.

### Nuclear protein extraction

Nuclear extracts from p53- and miR-34a-overexpressing cells were prepared as previously described [61]. p53-overexpressing cells were obtained by transfecting 8 µg of wild-type p53 (pCMV-p53wt).

### Electrophoretic mobility shift assay

Electrophoretic mobility shift assay (EMSA) was performed as previously described [62]. DNA probe and competitors were generated by hybridization with complementary single-stranded oligonucleotides and used in double-stranded form (supplementary Table 1). Briefly, 8 µg nuclear extracts were incubated with γ^32^P-labeled oligonucleotide probe (p53-cons) containing a p53 *consensus* binding site for 20 min. In competition assays, excess unlabeled oligonucleotide was pre-incubated for 30 min, prior to incubation with the probe p53-cons for additional 20 min. Super-shift reactions were performed with 1 µl of anti-p53 (DO-1 X, Santa Cruz Biotechnology), which was added to the reaction media after incubation of the probe. Protein-DNA complexes were resolved on 5% non-denaturing PAGE gels (29∶1 acrylamide∶bisacrylamide) in 0.5× Tris-borate buffer (45 mM Tris-borate, 1 mM EDTA). The gels were electrophoresed for 2.5 h, at 20 mA.

### Cytofluorometric analysis of apoptosis

Apoptotic cells were quantified by cytofluorometric analysis using a FACSCalibur (Becton Dickinson, Mountain View, CA) as described previously [63]. Cells were stained with the vital dye propidium iodide (PI; 5 µg/mL; Sigma, Steinheim, Germany) and concomitantly with Annexin-V-APC (eBioscience, Inc.), according to manufacturer's instructions, to determine phosphatidylserine exposure. Data were statistically evaluated using FlowJo software (Tree Star, Inc, Ashland, OR).

### Statistical Analysis

Results from different groups were compared using the Student's t test, or one-way ANOVA. Values of *p*<0.05 were considered statistically significant. All statistical analysis was performed with GraphPad InStat v3.05 software (GraphPad Software, Inc, San Diego, CA).

## Supporting Information

Table S1 **Sequence of oligonucleotides used in EMSA assay.** p53 binding motifs are shown in bold. p53 –cons, p53 *consensus* oligonucleotide; p53-cons-mut, oligonucleotide bearing a mutation in the consensus site; p53A and B, oligonucleotides containing either two or one quarter-sites known to be consensus sites for p53, repectively; NS, non-specific oligonucleotide.
(DOC)
