# Supplementary material for: miR-34a Regulates Mouse Neural Stem Cell Differentiation
Source: PLoS One. 2011 Aug 3;6(8):e21396. doi: 10.1371/journal.pone.0021396 (PMC3153928; doi:10.1371/journal.pone.0021396)
Supplement: Table S1 — Sequence of oligonucleotides used in EMSA assay. p53 binding motifs are shown in bold. p53 –cons, p53 consensus oligonucleotide; p53-cons-mut, oligonucleotide bearing a mutation in the consensus site; p53A and B, oligonucleotides containing either two or one quarter-sites known to be consensus sites for p53, repectively; NS, non-specific oligonucleotide. (DOC) [file pone.0021396.s001.doc]

| **Oligonucleotide** | **Sequence (5’→3’)** |
| --- | --- |
| p53-cons | AGCTT**AGACA**TGCCT**AGACA**TGCCTA |
| p53-cons-mut | ATGTTACACATTGCTACACATTGCTA |
| p53-A | **AGACA**TGCCT**AGACA**TGCCT |
| p53-B | ATGAATT**AGACA**GAATTAAT |
| NS | TCTCTCAGGCCTGCTGCTTAGGAGGCAACT |
